# Two-Photon-Excited Single-Molecule Fluorescence Enhanced by Gold Nanorod Dimers

**DOI:** 10.1021/acs.nanolett.2c01219

**Published:** 2022-05-16

**Authors:** Xuxing Lu, Deep Punj, Michel Orrit

**Affiliations:** Huygens-Kamerlingh Onnes Laboratory, Leiden University, 2300 RA Leiden, The Netherlands

**Keywords:** single-molecule fluorescence, two-photon excitation, plasmonic enhancement, gold nanorod dimer, single-molecule bursts, ATTO 610 dye

## Abstract

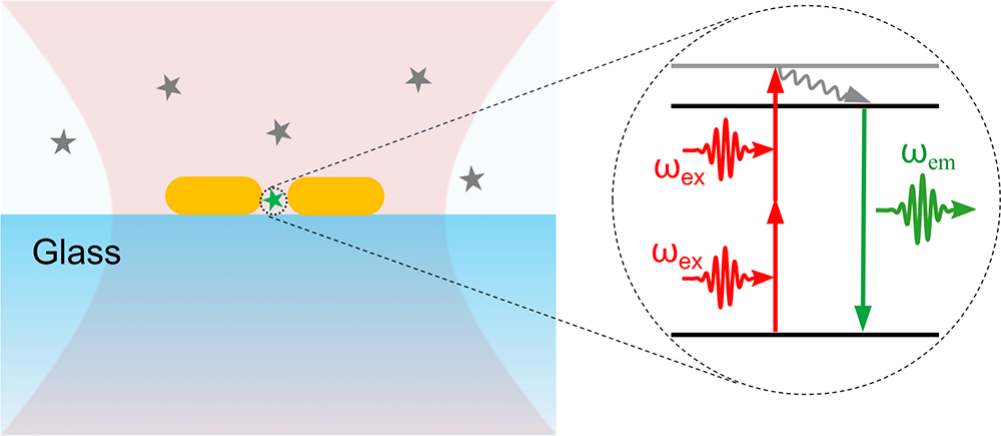

We demonstrate two-photon-excited single-molecule fluorescence enhancement by single end-to-end self-assembled gold nanorod dimers. We employed biotinylated streptavidin as the molecular linker, which connected two gold nanorods in end-to-end fashion. The typical size of streptavidin of around 5 nm separates the gold nanorods with gaps suitable for the access of fresh dyes in aqueous solution, yet small enough to give very high two-photon fluorescence enhancement. Simulations show that enhancements of more than 7 orders of magnitude can be achieved for two-photon-excited fluorescence in the plasmonic hot spots. With such high enhancements, we successfully detect two-photon-excited fluorescence for a common organic dye (ATTO 610) at the single-molecule, single-nanoparticle level.

## 1 Introduction

Two-photon-excited fluorescence is a nonlinear optical process, where a fluorophore simultaneously absorbs two photons of identical frequency to emit a photon with higher energy.^1,2^ Ever since its first prediction by Maria Goeppert-Mayer in 1931,^3^ and its experimental demonstration by Franken et al. and Kaiser and Garrett in the 1960s,^4,5^ two-photon excitation has attracted significant interest for its several advantages, such as strong background suppression,^1^ deeper tissue penetration,^6,7^ less photodamage to the samples,^8,9^ and intrinsic optical sectioning.^10−12^ Practical applications of two-photon excitation, however, are limited by the requirement of extremely high photon density. The invention of ultrashort-pulse lasers has led to a rapid growth of two-photon-based techniques in various scientific fields,^10^ such as imaging,^6,7,10−12^ photodynamic therapy,^13^ microfabrication,^14−18^ or optical storage.^19−23^

Recent progress in nano-optics has made it possible to enhance the two-photon-excitation process through near-field confinement of the excitation field by plasmonic nanostructures.^24,25^ The local electromagnetic field around the plasmonic structures can be enhanced^26^ by one or more orders of magnitude, depending on their shapes, sizes, and the materials.^27−29^ Among all kinds of plasmonic structures, wet chemically synthesized gold nanorods have been widely exploited in the context of field-enhanced spectroscopy, mainly due to their high single-crystalline quality,^30,31^ facile synthesis compared to other fabrication techniques,^32,33^ and narrow tunable plasmon resonances.^34,35^ In the past few years, gold nanorods have been applied to enhance the fluorescence of single weak emitters.^36−39^ Fluorescence enhancements by 3–4 orders of magnitude have been reported with single gold nanorods (GNRs) of suitable plasmon resonances, through enhancement of both the excitation and radiative rates of the emitters.^36−39^ Higher enhancement factors of about 10^5^ to 10^6^ have also been achieved in strongly coupled gold nanosphere dimers.^40^

Fluorescence upon two-photon excitation is expected to give rise to much larger enhancement than one-photon-excited fluorescence due to the quadratic dependence of this process on the excitation intensity.^41−45^ The effective enhanced volume under two-photon excitation is restricted to much smaller regions, which improves the selectivity of detecting the enhanced two-photon-excited fluorescence signals against background. Single-molecule or single-particle detection of two-photon-excited fluorescence opens up the door to reveal the intrinsic nature of nonlinear interaction between photons and molecules,^46^ which is usually hidden in ensemble experiments. By using a single GNR, our group has earlier reported strong two-photon-excited photoluminescence enhancement of single colloidal quantum dots (Qdots), with enhancement factors larger than 10,000-fold.^44^ Such high enhancement, however, is still not enough to distinguish the enhanced two-photon-excited fluorescence of single organic molecules from background because molecules have much lower two-photon absorption cross-section than Qdots (about 1,000 times smaller). It is not clear that the single-molecule regime can be reached at all because of two main reasons. First, two-photon excitation might not be efficient because of an unfavorable photon budget and of the huge enhancement factor required. Although two-photon-excited emission by down to about 20 molecules has been reported,^45^ bleaching or blinking of the molecules might limit the signal-to-noise ratio of single-molecule emission. Second, plasmonic enhancement with light pulses might be intrinsically limited by the thermal reshaping of the structures and/or by the transient broadening of plasmon resonances in pulsed light.^47^ These effects could limit the maximum power of the laser pulses to such low levels that single-molecule detection would be impossible in practice. A few other plasmonic structures, such as nanofabricated bow-tie antennas,^43^ have also been reported to enhance two-photon excitation, yet the enhancement factors were even lower than those of single isolated GNRs. Ojambati et al. have recently demonstrated that two-photon absorption of ruthenium-bipyridinium complexes can be enhanced by 10^8^ inside the nanoparticle-on-mirror (NPoM) sub-nanometer cavities.^45^ NPoM are very convenient nanostructures^48^ providing large enhancement factors with a capability of even detecting single diffusing molecules.^49^ Yet, despite an extremely large enhancement factor, the practical uses of two-photon-excited fluorescence by NPoM structures would be limited by the restricted diffusion to replace a dye molecule after photobleaching, and by the steric hindrance of a carrier biomolecule, for example, that would not fit into the compact and closed design of a NPoM cavity. Therefore, it is interesting to look for two-photon-excited fluorescence of single molecules in more open plasmonic cavities.

The present work intends to answer the question: Does thermal reshaping of gold nanostructures or transient broadening of the plasmon prevent the detection of single molecules by two-photon-excited fluorescence? We demonstrate two-photon-excited fluorescence enhancement experiments on single organic fluorophores using end-to-end assembled GNR dimers. The end-to-end assembly was achieved by tip-specific functionalization of the GNRs with molecular linkers. We employed molecular linkers based on biomolecule pairs consisting of two biotin disulfides bridged by a streptavidin, which ensured an open cavity for single-molecule detection with an interparticle gap of around 5 nm.^50^ We choose this comparatively large gap size, at the cost of a reduced enhancement, to respond to two requirements: (i) to keep the plasmonic cavity open enough to accommodate possibly large biomolecules and (ii) to avoid complications from surface-enhanced Raman scattering (SERS) signals or from metal luminescence bursts that are sometimes observed in cavities with gaps smaller than 1–2 nm.^51−53^ We applied the GNR dimers to enhance the two-photon-excited fluorescence of commercial organic dyes, which have a broad two-photon absorption band in the near-infrared range. Theoretical simulations indicate that two-photon-excited fluorescence enhancement of these molecules can exceed 10^7^ in the gaps between the GNRs. With such high enhancement factors, we succeeded in detecting enhanced two-photon-excited fluorescence from single molecules.

## 2 Results and Discussion

Supporting Information Figure S1 schematically illustrates our approach of GNR dimers synthesis. Briefly, a GNR colloid solution (simplified as GNR solution in the following) was mixed with the pretreated biomolecular linkers that consisted of at least two biotin disulfides linked with one streptavidin. These molecular linkers were bound to the tips of the GNR through thiol attachment in the presence of cetyl(trimethyl)ammonium bromide (CTAB), which occupied more compactly the sides of GNRs, leaving only the tips reactive for the linkers. Therefore, the GNRs were linked by the molecular linker at the ends, leading to the end-to-end assembly of GNRs in the solution (see Figure S1a). We monitored the assembly process by measuring the real-time absorption spectrum of the mixture to ensure most assembled structures to be the end-to-end dimers.^54^ A small amount (10 μL) of the assemblies of GNRs were deposited from the solution onto a clean cover glass slide. We stop the assembling by covering the slide with the assembly solution with another clean slide (see Figure S1b,d). By using this strategy, a very thin film of the assembly solution is formed between the two slides, which dry very quickly, leaving every assembled GNRs stuck on either one of the glass slides. We performed UV/Ozone cleaning to remove all of the organic molecules around the GNRs, to ensure the proper binding of the GNRs on the glass surface, and to create empty gaps between the GNRs of the assemblies.

We performed two-photon-excited single-molecule fluorescence experiments on a commercial dye called ATTO 610 (from ATTO-TEC) enhanced by the GNR dimer structures. ATTO 610 is a bright dye with high photostability under one-photon excitation. It has a one-photon absorption maximum at 616 nm and an emission maximum at 633 nm. ATTO 610 also exhibits two-photon absorption in the near-infrared range under illumination with ultrashort pulses. In our experiment, a mode-locked Ti:sapphire laser (Coherent Mira 900) with a pulse width of ∼220 fs was used as the two-photon-excitation source. The experiment was performed on a home-built confocal microscope. Circular polarization was used to enable the excitation of all of the GNR dimers irrespective of their random orientations in the focal plane.

**Figure 1:**
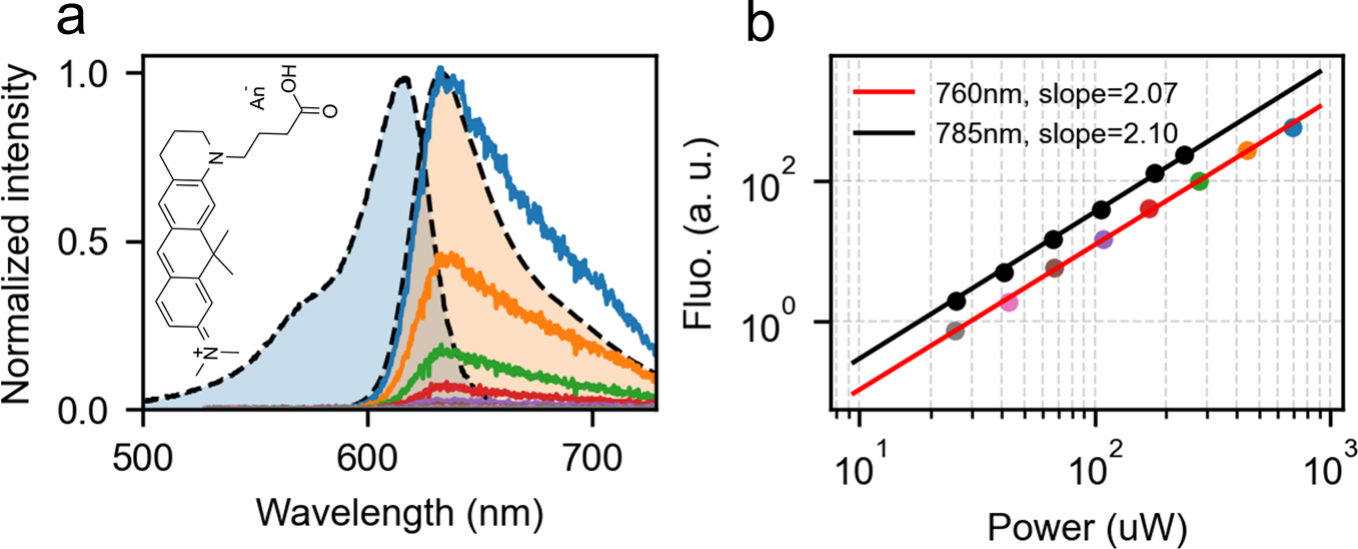
Optical characterization of ATTO 610. (a) Normalized one-photon absorption (blue-shaded band with dashed outline) and emission (light orange-shaded band with dashed outline) spectra of ATTO 610, respectively. The solid lines give the emission spectra of 4 μM ATTO 610 excited by ∼220 fs laser pulses at 760 nm for different powers depicted in panel b as matching dot colors for 760 nm wavelength. The integrating time for recording the spectra was set as 120 s. The inset shows the chemical structure of ATTO 610. (b) Power dependence of the emission integrated over wavelengths ranging from 555 to 728 nm, excited at the wavelength of 760 nm (dot colors correspond to the spectra colors in panel a) and 785 nm (black dots). The power law fits (solid lines) show close-to-perfect quadratic dependence of the emission on the excitation power for both excitation wavelengths.

By illuminating the aqueous solution of ATTO 610 with the femtosecond laser at different wavelengths in the infrared, we got very faint fluorescence signals with spectral shape similar to the fluorescence of ATTO 610 under one-photon excitation. Notably, due to the large energy gap between the excitation laser of 760 nm and the excited state of ATTO 610, one-photon excitation of this molecule by an anti-Stokes or hot-band process is not possible. To verify that the emission indeed stems from two-photon excitation, we collected the emission spectra with respect to the excitation intensity. Figure 1a illustrates the power dependence of the emission spectra at the excitation wavelength of 760 nm. As is shown in Figure 1a, the emission spectra show little change in the shape but the intensities decrease dramatically as we reduce the excitation power. As is depicted in Figure 1b, the integrated intensities of the emission spectra, for both excitation wavelengths of 760 and 785 nm, show a close-to-perfect quadratic dependence on the excitation power, confirming that the observed fluorescence arose from two-photon excitation for both excitation wavelengths. The broad TPA band of ATTO 610 offers us the flexibility of optimizing the collected signals by tuning the excitation wavelength and the plasmon resonance of the GNR dimers.

We performed single-molecule experiments on the assembled GNR dimers immobilized on a clean glass cover slide. The GNRs were immobilized on the glass by strong van der Waals forces after removal of all organic molecules through ozone/UV cleaning. A cross was scratched on the slide as a marker to locate the positions of the observed particles for further study. In the experiments, we chose commercial GNRs (NR-40-700, NanoSeedz) as the building blocks of the assemblies. These rods have an average diameter of 40 nm and a longitudinal plasmon mode at the wavelength of 700 nm. Simulations predict the bright plasmon mode of the end-to-end gold nanorod to red shift to ∼760 nm after removing all of the organic molecules, if we assume the interparticle separation of the dimer to be ∼5 nm, considering the molecular size of streptavidin.^50^ Thereafter the laser wavelength of 760 nm was used as the excitation to ensure maximum enhancement. After the optical measurements, SEM images were taken to examine the structural details of the measured assemblies.

**Figure 2:**
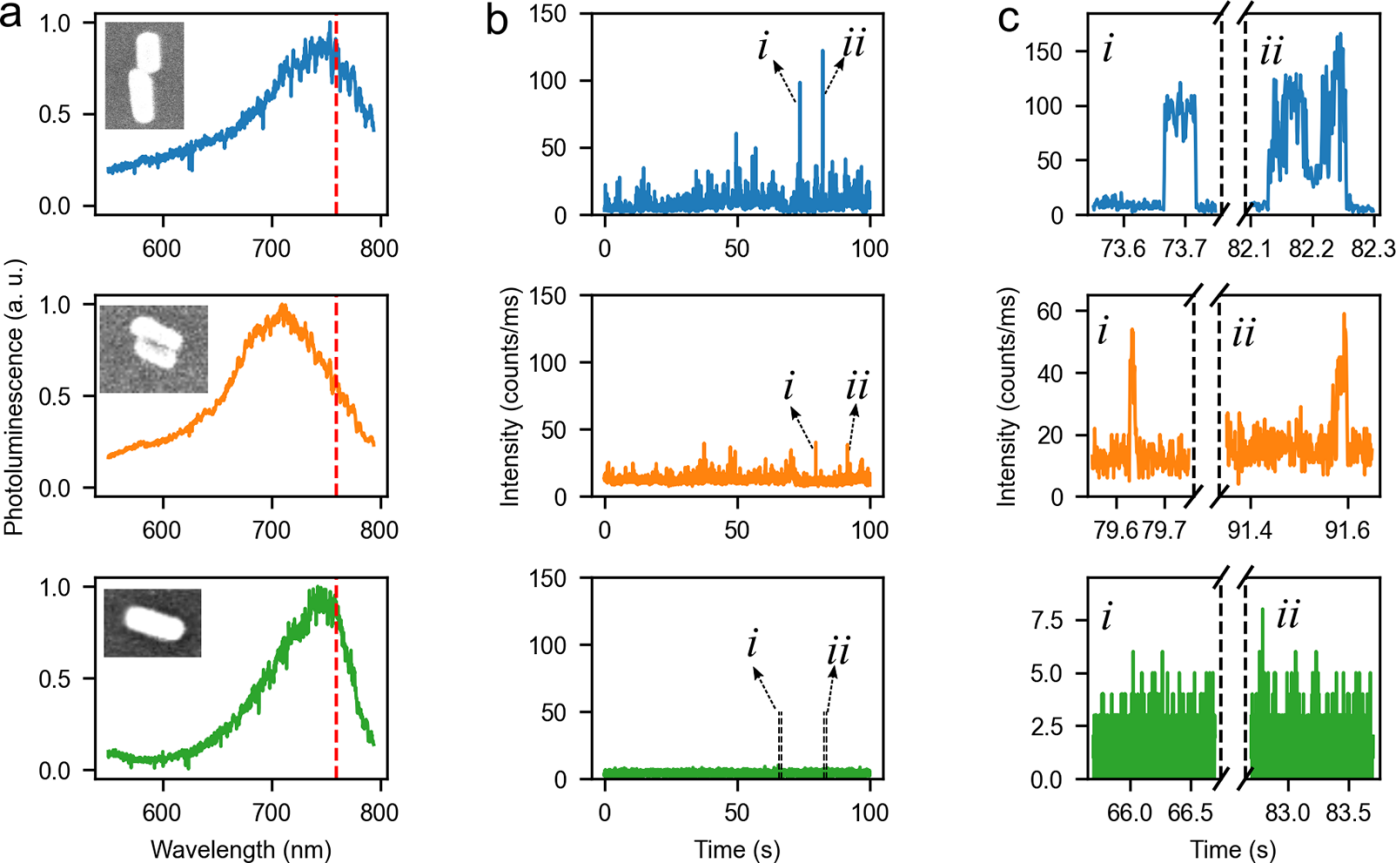
Two-photon-excited single-molecule fluorescence enhancement. (a) One-photon-excited luminescence spectrum taken on three different structures made of gold nanorods: end-to-end dimer, side-by-side dimer, and a single gold nanorod, acquired under excitation by a circularly polarized 532 nm CW laser. Inset shows the SEM images of the structures. (b) Respective intensity traces taken in the presence of 20 nM ATTO 610 dyes, excited by a femtosecond laser at the wavelength of 760 nm and at the power of ∼2 μW. The binning time was set as 10 ms. (c) Respective zoomed-in views of the photoluminescence intensities indicated by the arrows shown in panel b. The binning time for the zoomed-in time traces was set as 1 ms. The single-step changes of those bursts in the time traces from the GNR dimers (blue and orange) confirm that the enhanced fluorescence signals are stemming from single molecules. As expected, the green trace of a single GNR does not show any clear bursts (see zoomed-in traces at two randomly chosen points).

Figure 2 illustrates typical single-molecule measurements of the two-photon-excited fluorescence enhanced by GNR assemblies. We first recorded the photoluminescence of each particle under excitation with the femtosecond laser, in the presence of 20 nM ATTO 610 molecules. A short-pass filter (Fluorescence Edge Filter 745/SP, BrightLine) was used to reduce intrinsic luminescence background from the gold particles. The particles showing intensity bursts in these luminescence time traces were supposed to be the assembled structures. Indeed, simulations and further experiments both show that, with enhancement by a single GNR, it is very difficult to extract single-molecule fluorescence from the luminescence background of the nanorod under two-photon excitation. After rinsing the sample with clean water several times, we measured the one-photon-excited luminescence spectra of the particles to determine their plasmon resonances. With the help of the marked cross, we further identified the structures of these gold nanorod assemblies by comparing the SEM image with the scatter image, as one can see from the example in Figure 2a.

We first compared two-photon-excited fluorescence enhancement on two typical GNR dimers, assembled in either the end-to-end or the side-by-side configuration. As is shown in Figure 2a, for the end-to-end dimers, the plasmon resonance was red-shifted to wavelengths around 760 nm as a result of the longitudinal plasmonic coupling of each rod, while, for the side-by-side structure, the plasmon resonance showed little change compared to the single GNRs. As is depicted in Figure 2b, for both side-by-side and end-to-end dimers, we observed intensity bursts in the luminescence time traces. From the comparison of the maximum intensity bursts in Figure 2b,c, we can see that the end-to-end dimer gives much larger two-photon fluorescence enhancement than the side-by-side dimer, which is due to the stronger plasmon coupling of the GNRs and stronger near-field confinement in the end-to-end configuration. We also notice that, as a result of the plasmon red shift, the remaining luminescence background of end-to-end dimers, after passing through the short-pass filter, was reduced more efficiently than the background of the side-by-side structure, which may further improve the single-molecule detection sensitivity of the two-photon-excited fluorescence. As a comparison, we also performed the measurements on the single GNRs. We selected longer GNRs, resonant with the laser wavelength at 760 nm. A typical measurement on a single GNR is shown in Figure 2 (green line). From Figure 2b (green), we do not see any signal burst in the time trace, which indicates that the enhancement by a single GNR is too weak for us to detect single-molecule fluorescence under two-photon excitation.

By analyzing the strongest bursts in the fluorescence time trace enhanced by the end-to-end dimer (Figure 2c), we see typical single-step single-molecule bursts with a time duration in the order of 10 ms, which confirm that the enhanced fluorescence signals are stemming from single molecules. Figure S6 in the Supporting Information shows the study of the dependence of the fluorescence burst frequency on the concentration of ATTO 610 dye. We confirmed that the burst frequency scales linearly with the concentration. This provides further evidence that we are seeing single-molecule fluorescence signals.

**Figure 3:**
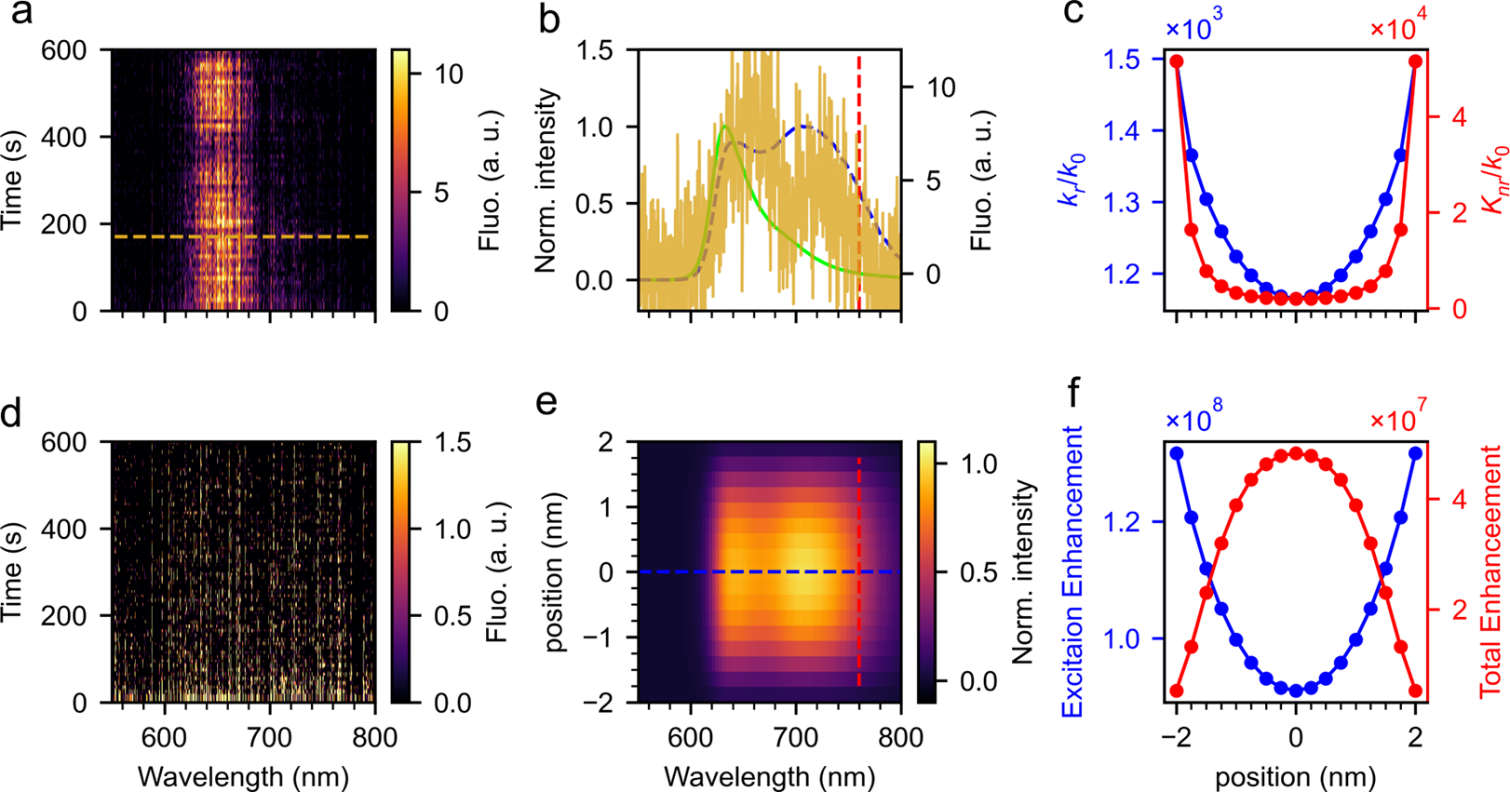
Two-photon-excited fluorescence enhancement in the plasmonic hot spot of an end-to-end gold nanorod dimer. (a and d) Real-time spectra of a gold nanorod dimer with (a) and without (d) the presence of 100 nM ATTO 610 in solution. (b) Comparison of the measured spectrum (orange, corresponding to the recorded time of the orange dashed line in panel a) with the spectrum of free ATTO 610 dye in solution (green solid) and the simulated enhanced spectra (blue dashed, corresponding to the blue dashed line in panel e) in the hot spot. (e) Simulated emission spectra of an ATTO 610 molecule at different positions along the main axis of the gold nanorod dimer in the gap. (c) Calculated radiative (blue) and nonradiative (red) enhancement factor as functions of the position in the hot spot and (f) respective excitation (blue) and total emission (red) enhancements. The vertical red dashed lines in panels b and e represent the wavelength of the femtosecond laser.

Before attributing the intensity bursts to the fluorescence from single ATTO 610 molecules enhanced inside the plasmonic hot spot, we monitored the real-time spectra on the particle excited by the femtosecond laser, with/without the presence of 100 nM ATTO 610 in solution. Figure 3a shows one case of the real-time recording of the plasmon-enhanced two-photon-excited single-molecule emission spectra. The spectra were recorded in a time series of 10 min with spectral acquisition time of 10 s for each step. In the measurement, we kept the excitation power as low as possible (∼0.5 μW) to reduce the luminescence background from gold particles. Indeed, higher powers cause heating of the molecule and of the nanorod dimer and thereby lead to anti-Stokes emission, including hot-band fluorescence, hot-band photoluminescence, and anti-Stokes SERS. From Figure 3a, we can clearly see the emission pattern between 620 and 690 nm, with the intensities fluctuating over time. Such an emission pattern is removed upon replacing the ATTO 610 solution by clean water, as is shown in Figure 3d, which demonstrates that the signals were from ATTO 610 molecules near the particle. The spectral range, shown in Figure 3a, was noticeably wider compared to the emission of free dyes in solution (green solid line in Figure 3b), mainly due to coupling of the molecules with the plasmonic modes.

Specifically, we compared one recorded spectrum (corresponding to the orange dashed line in Figure 3a) with the spectrum of the free dye (green line) in Figure 3b. The result shows drastic changes in spectral shape for the emission enhanced by the plasmoic nanoresonator, as we can clearly see a second peak at the wavelength around 730 nm in the enhanced spectrum (orange line). This spectral reshaping can be explained by the well-known effect that the far-field emission of an emitter can be modified by coupling with a plasmonic structure,^55,56^ through (i) the Purcell effect that enhances the spontaneous emission rate and (ii) the nonradiative dissipation loss inside the metal that quenches the emission. For comparison, we show in Figure S9c the measured spectrum averaged over time, which does not show the 730 nm peak. This disappearance may be due to averaging over many different molecules having different positions or orientations in the gap of the gold nanorod dimer. Here, we employed a simple radiative dipole model to investigate the influence of the GNR dimer on the emission of ATTO 610 molecules. For the sake of simplicity, we considered the dimer consisting of two identical GNRs with the longitudinal axes oriented in parallel and separated by a gap of 5 nm. More details about the simulations are given in the Supporting Information.

From Figure 3b, we see a good agreement between the spectral shapes of the measured emission (orange) and the simulated emission calculated at the position shown in Figure 3e (blue dashed). The increase of the emission rates in the band of longer wavelength, therefore, can be attributed to the selective enhancement of the vibrational subbands in resonance with the plasmon modes. From the simulations, we also notice that the nonradiative dissipation dominates the decay rates of the excited molecule inside the gap (Figure 3c), which quench the emission rates by a factor of about 2 in the center of the dimer (Figure 3f). As the molecule moves closer to the gold surface, the nonradiative rate increases more than the excitation and radiative enhancements, which further reduces the fluorescence enhancement, as is shown in Figures 3c,f. As the simulation of Figure 3f shows, the excitation rate enhancement is 10^8^ times, the radiative rate enhancement is about 1,000 times, and the emission reduction due to the nonradiative process is by around 2,000 times. As a consequence, we see a maximum enhancement factor of about 5 × 10^7^ for the two-photon-excited fluorescence at the center of the dimer.

**Figure 4:**
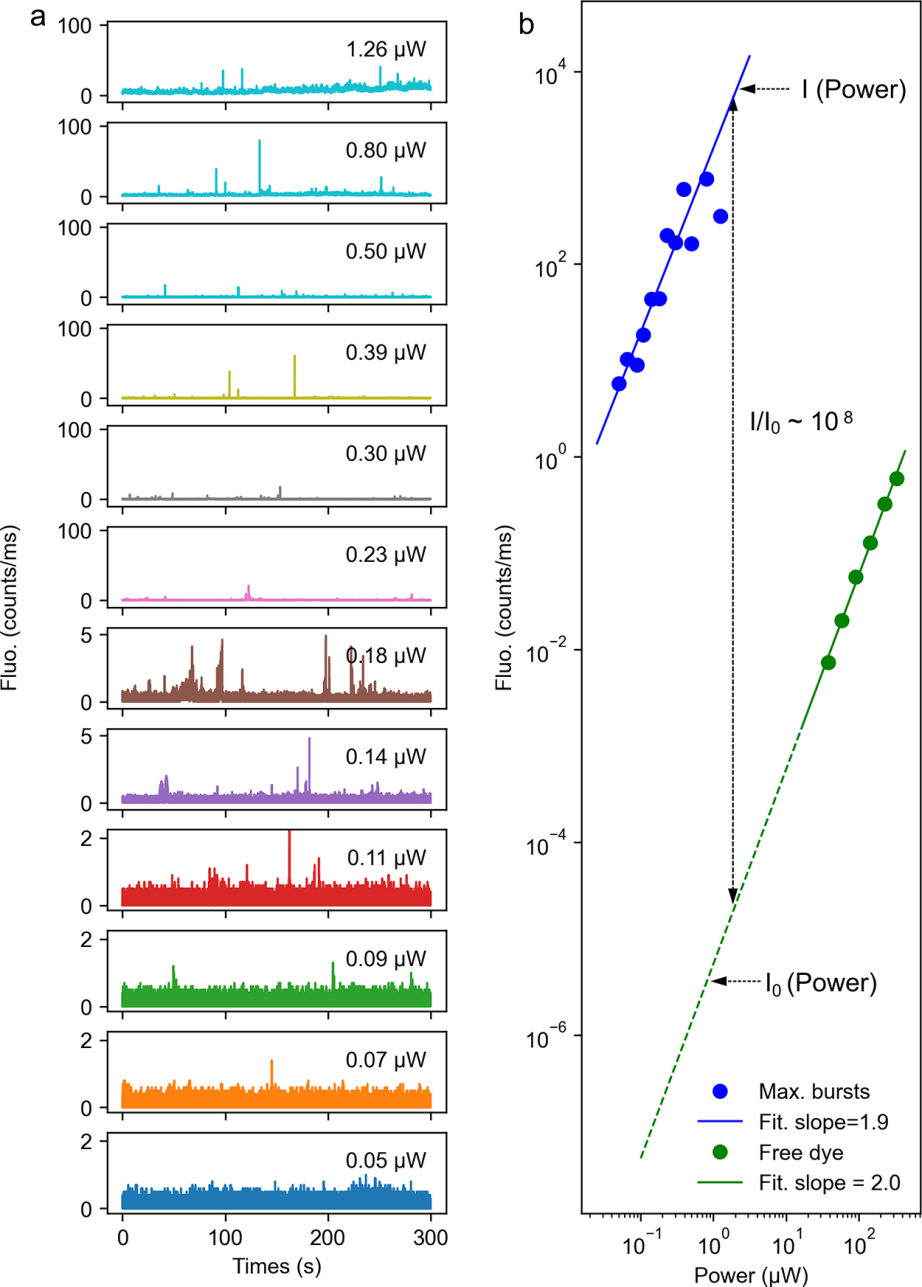
Power dependence of the emission. (a) Emission time trace (10 ms/bin) as a function of excitation power recorded on a gold nanorod self-assembled nanostructure. The particle was immersed in a solution of ATTO 610 with the concentration of 30 nM. (b) Power dependence of the maximum fluorescence burst intensity (blue) and the averaged unenhanced fluorescence per molecule.

To confirm that the observed emission indeed stems from two-photon excitation, we performed a power dependence measurement on an individual GNR self-assembled nanostructure that gave single-molecule bursts. Time traces with intensity bursts excited at different powers are displayed in Figure 4a. As expected, the fluorescence signals, represented by intensity bursts of the time traces, tend to increase as we raise the excitation power. We provide zoomed-in views of the largest bursts of these time traces in the Supporting Information (Figure S8), all of them showing single-step changes and confirming that the enhanced fluorescence signals are from single molecules. A comparison of the maxima of the fluorescence bursts excited at different powers is shown in Figure 4b. (blue dots). These maximum intensity bursts were supposed to be the signals from the molecules at the best position of enhancement. In real experiments, however, the molecules can approach or stick to the glass surface at any position with random orientations inside the plasmonic hot spot. The enhancement factor for the molecules can therefore vary quite randomly, resulting in a random distribution of intensity bursts in each time trace. As a consequence, we cannot ascertain that we measure the largest enhancement factor within our acquisition period (here 300 s) and one can expect that a longer time trace should lead to a larger maximum burst intensity. However, earlier studies of enhanced one-photon-excited fluorescence^57^ have shown that this effect is relatively minor on the log scale employed. We assumed that this rule also holds for two-photon-excited fluorescence. We find a quasi-quadratic dependence on the excitation power for the ”cherry-picked”^57^ maximum fluorescence bursts (blue line with the fitting slope of 1.9 in Figure 4b), which indicates that the enhanced signals were indeed from molecules excited by two photons.

In order to calculate the enhancement factor of the two-photon-excited fluorescence, we compared the enhanced burst intensities with the unenhanced signals from a molecule in solution. We performed power dependence measurements for ATTO 610 molecules in the solution (3 μM) to estimate the two-photon-excited fluorescence signals excited at low powers where the unenhanced signal of a single molecule in the solution is too weak to be detected. As shown in Figure S4b, the averaged intensity of the fluorescence time trace measured in the solution (Figure S4a) also scales quadratically with the excitation power. By taking account of the number of molecules in the focal volume, we show the quadratic dependence of the averaged fluorescence per molecule on the excitation power (green line) in Figure 4b (for details see the Supporting Information). By scaling the unenhanced fluorescence quadratically with the excitation power, we can estimate an enhancement factor of up to ∼10^8^ for the two-photon-excited fluorescence.

## 3 Conclusion

In summary, we have demonstrated the single-molecule detection of two-photon-excited fluorescence via the enhancement by self-assembled GNR dimers. In the experiment, we control the interparticle gaps by exploring the streptavidin-biotin disulfides as molecular linkers, which separate the nanorods by a distance of about 5 nm. Correlated scanning electron microscope images were taken later to examine the configurations of the GNR dimer structures (see the Supporting Information). Theoretical results indicate that two-photon-excited fluorescence rates can be enhanced by a factor of up to 10^7^ to 10^8^. Motivated by such high theoretical enhancement factor, we were able to detect the two-photon-excited fluorescence from single ATTO 610 dyes with an enhancement factor of ∼10^8^. This large enhancement is the result of plasmonically enhanced strong near field in the gap of the gold nanorod dimer along with the quadratic dependence of two-photon absorption and excitation intensity. Our results show that gold nanorod dimers can be excellent nanostructures to explore two-photon-based fluorescence applications.

## Supporting Information Available

Assembly of gold nanorods, deposition of gold nanorod assemblies, two-photon microscopy, two-photon-excited fluorescence of ATTO 610 in solution, and numerical simulations of two-photon-excited fluorescence enhancement (PDF)
