# Supplementary material for: Two-Photon-Excited Single-Molecule Fluorescence Enhanced by Gold Nanorod Dimers
Source: Nano Lett. 2022 May 16;22(10):4215–22. doi: 10.1021/acs.nanolett.2c01219 (PMC9136919; doi:10.1021/acs.nanolett.2c01219)
Supplement: Supplementary file 1 — nl2c01219_si_001.pdf [file nl2c01219_si_001.pdf]

Supporting Information: Two-photon-excited single-molecule fluorescence enhanced by gold nanorod dimers

Xuxing Lu, Deep Punj, and Michel Orrit*

Huygens-Kamerlingh Onnes Laboratory, Leiden University, 2300 RA Leiden, Netherlands

E-mail: Orrit@physics.leidenuniv.nl

Contents

Assembly of gold nanorods S-3
Deposition of gold nanorod assemblies S-4
Two-photon microscopy S-7
Two-photon-excited fluorescence of ATTO 610 in solution S-7
Numerical simulations of two-photon-excited fluorescence enhancement S-14

References **S-17**

Assembly of gold nanorods

The GNRs were self-assembled in an end-to-end fashion through the molecular linkers based on the specific molecular recognition of biotin and streptavidin. For the assembly, a biotin disulfide solution (20 μM), EZ-link Biotin HPDP (Pierce), was pretreated with a reducing agent solution, TCEP (tris(2-carboxyethyl)phosphine), with a 1:10 biotin/TCEP ratio. This reaction was allowed at room temperature for 15 min to break the disulfide bonds in the Biotin HPDP molecules. The mixture solution was added to the streptavidin solution (1 μM) in phosphate buffered saline solution (PBS, pH = 7.4), with a ratio of 4:1 for biotin/streptavidin. The incubation lasted for 45 min to allow the binding of streptavidin with at least two biotins. The excess unbound biotin disulfide and TCEP were removed by centrifugation in centrifugal filter devices (Ultra-0.5 10K, Amicon). The residue was dispersed in 100 μL deionized water. A commercially available GNR solution (NA-40-700, OD-50, Nanoseedz) was diluted to the desired concentration according to its optical density (OD) of ~ 0.3. The GNR solution had plasmon absorption at 700 nm, and the average diameter of the GNRs was 40 nm. 40 μL of a biotin-streptavidin solution was added into 500 μL as-prepared GNR solution to trigger the end-to-end assembly of the GNRs. The assembly of GNRs was monitored and recorded by a Cary 50 UV-Vis spectrometer (Varian Inc. Agilent Technology, USA) every 2 min. Figure S2 shows the evolution of the measured extinction spectra. Over time, the intensity of the longitudinal SPR peak (around 700 nm) decreases gradually without any spectral shift and the red-side shoulder becomes broader, indicating the end-to-end assembly of GNRs in the solution. From the spectra, we conclude that the fraction of the longer chain assemblies (e.g. the assemblies with more than three GNRs) in the solution is low, as the extinction at the end of the longer-wavelength tail is relatively small.[S1] We also notice no change in the shape of the transverse SPR band except for a reduction in the intensity. This indicates that the proportion of side-by-side assemblies of GNRs is very low. The ratio of short-chain assemblies (e.g. GNR dimers) can be further controlled by adjusting the assembly time and the stoichiometric ratio between GNR and

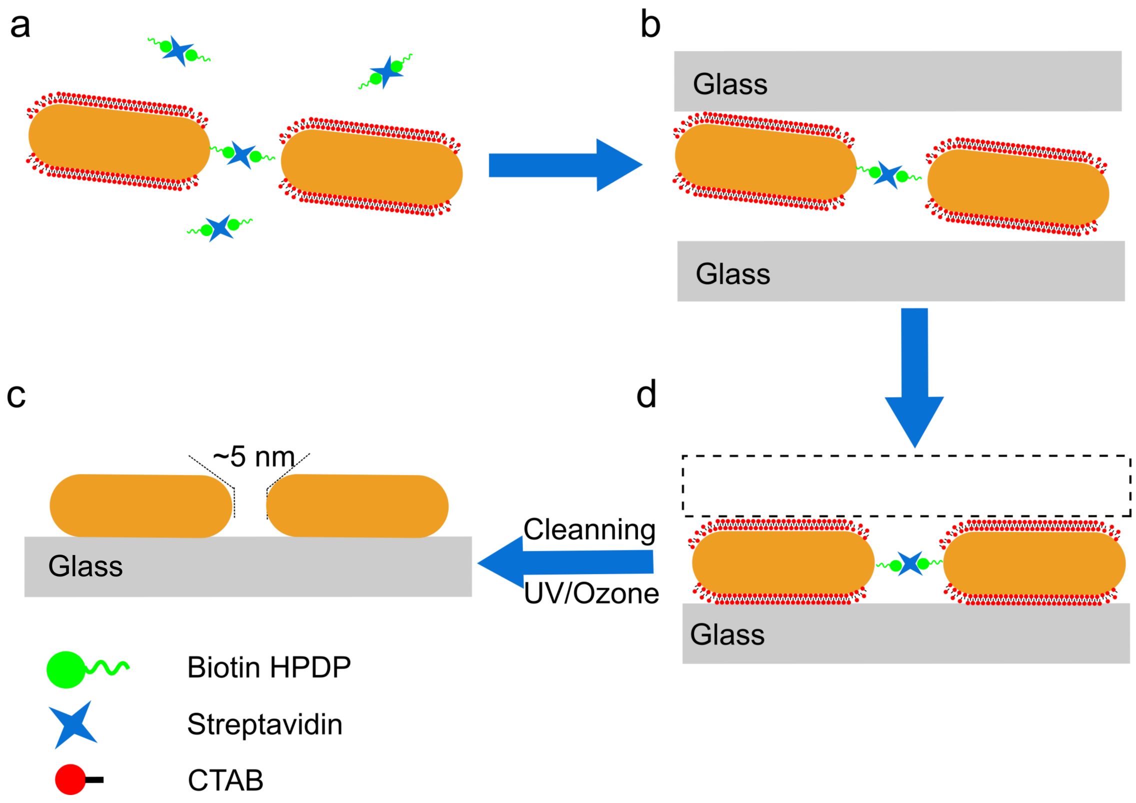

Figure S1: Scheme for end-to-end assembly of gold nanorod dimers. (a) Pre-treated molecular linkers based on biotin disulfide-streptavidin biomolecular pairs were added to the GNRs solution to trigger the end-to-end assembly of GNRs. (b,d) The assembled GNR dimers were deposited onto a clean glass, which was covered by another glass slide to stop the assembling of GNRs. (c) UV/Ozone cleaning was performed to remove the organic molecules around the GNRs.

molecular linker.

Deposition of gold nanorod assemblies

When the extinction peak of the solution dropped by a ratio of 1/4, 10 μL of the GNR assembly solution was deposited onto a clean cover glass slide (Menzel-Gläser, $\phi = 25$ mm, No. 1) with a scratched cross, and was immediately covered with a second glass slide. The capillary action between the two slides squeezed the assembled solution and formed a thin layer. This strategy helps stop further assembly of GNR, and deposits the GNR assemblies uniformly on the surfaces of the glass slides. After deposition, the two slides were separated by immersion into clean deionized water. After that, we performed UV/Ozone cleaning to

remove all the organic molecules around the GNRs and to ensure the proper sticking of the GNRs on the glass surface, and to create free gaps between the GNRs of the assemblies. Figure S3 shows typical SEM images of such an assembly sample. These SEM images of GNR assemblies are taken from the same sample at different areas. GNRs dimers are highlighted by green circles. We can see that the yield of end-to-end GNRs dimers can be close to 50%.

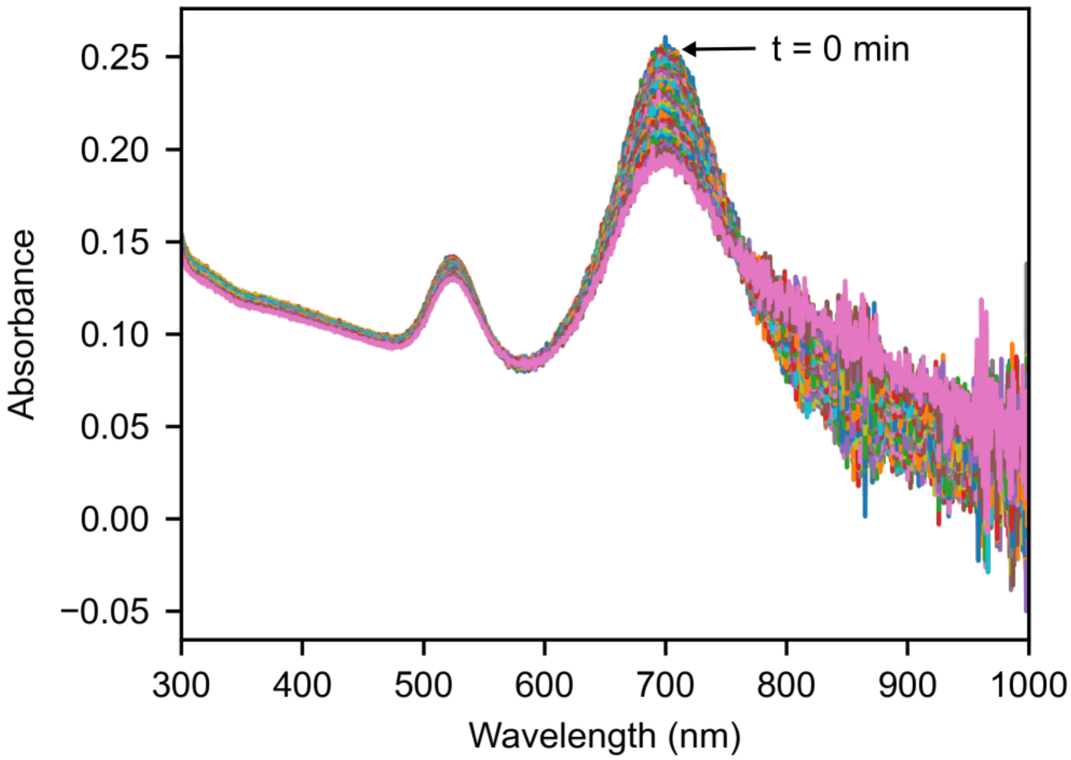

Figure S2: Evolution of the extinction spectra of a suspension of gold nanorods in the presence of biotinylated streptavidin. The spectra were recorded every 2 min.

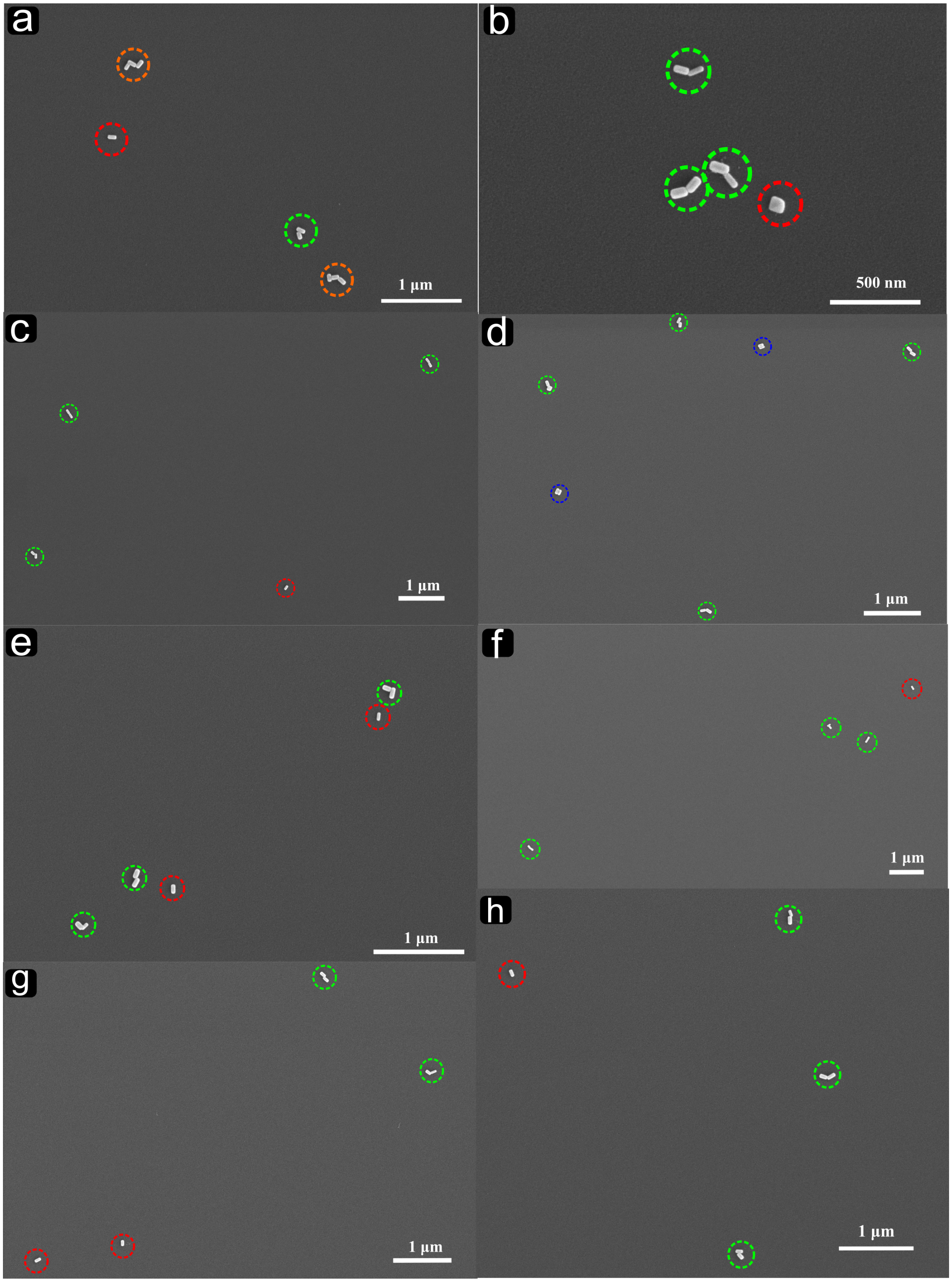

Figure S3: SEM images of GNR assemblies of different areas from the same sample. The assemblies of different morphologies are highlighted by the colored circles: end-to-end GNR-dimer: green, single GNR: red, side-by-side GNR dimer: blue, and clusters: orange

Two-photon microscopy

We performed two-photon-excited fluorescence measurements on a home-built confocal microscope. A mode-locked titanium-sapphire laser (Coherent Mira 900), with a pulse repetition rate of 76 MHz and pulse width of $\sim$ 220 fs, was used as the two-photon excitation source. Circular polarization was used in the whole study, as it can efficiently excite all gold nanorod assemblies, regardless of their random orientations in the focal plane. The light source was focused by an oil immersion objective with a numerical aperture (NA) of 1.4. A short-pass filter (Fluorescence Edge Filter 745/SP, BrightLine) was used to separate the fluorescence signal from the background of scattered laser light. To get the one-photon-excited photoluminescence spectrum of each gold nanorod assembly, a 532 nm continuous-wave laser was used as the excitation source, and the spectrum was recorded with a liquid-nitrogen-cooled spectrometer (Acton SP-500i, Princeton Instruments). Fluorescence time traces were recorded with an avalanche photodiode (SPCM-AQRH-16, Excelitas) and processed with a time-correlated single-photon counting (TCSPC) card (TimeHarp 200, PicoQuant GmbH).

Two-photon-excited fluorescence of ATTO 610 in solution

We first recorded the emission spectra from the ATTO 610 solution ($\sim$ 4 μM) excited at different laser powers. The exposure time was set as 120 s for each spectrum. In the study, we compared the power-dependent emission of ATTO 610 for two different excitation wavelengths 760 nm and 785 nm, while keeping the width and repetition rate of the pulse constant. For both wavelengths, the integrated intensity (wavelength range from 555 nm to 728 nm) depends quadratically on the excitation power.

To estimate the average fluorescence rate of individual ATTO 610 molecules without enhancement, we performed power-dependent measurements on an ATTO 610 solution with the concentration of 3 μM. Due to the extremely weak two-photon excitation efficiency of these molecules, much higher laser power was required to get enough signal from the solution, compared to enhanced experiments. To get rid of the unavoidable background of

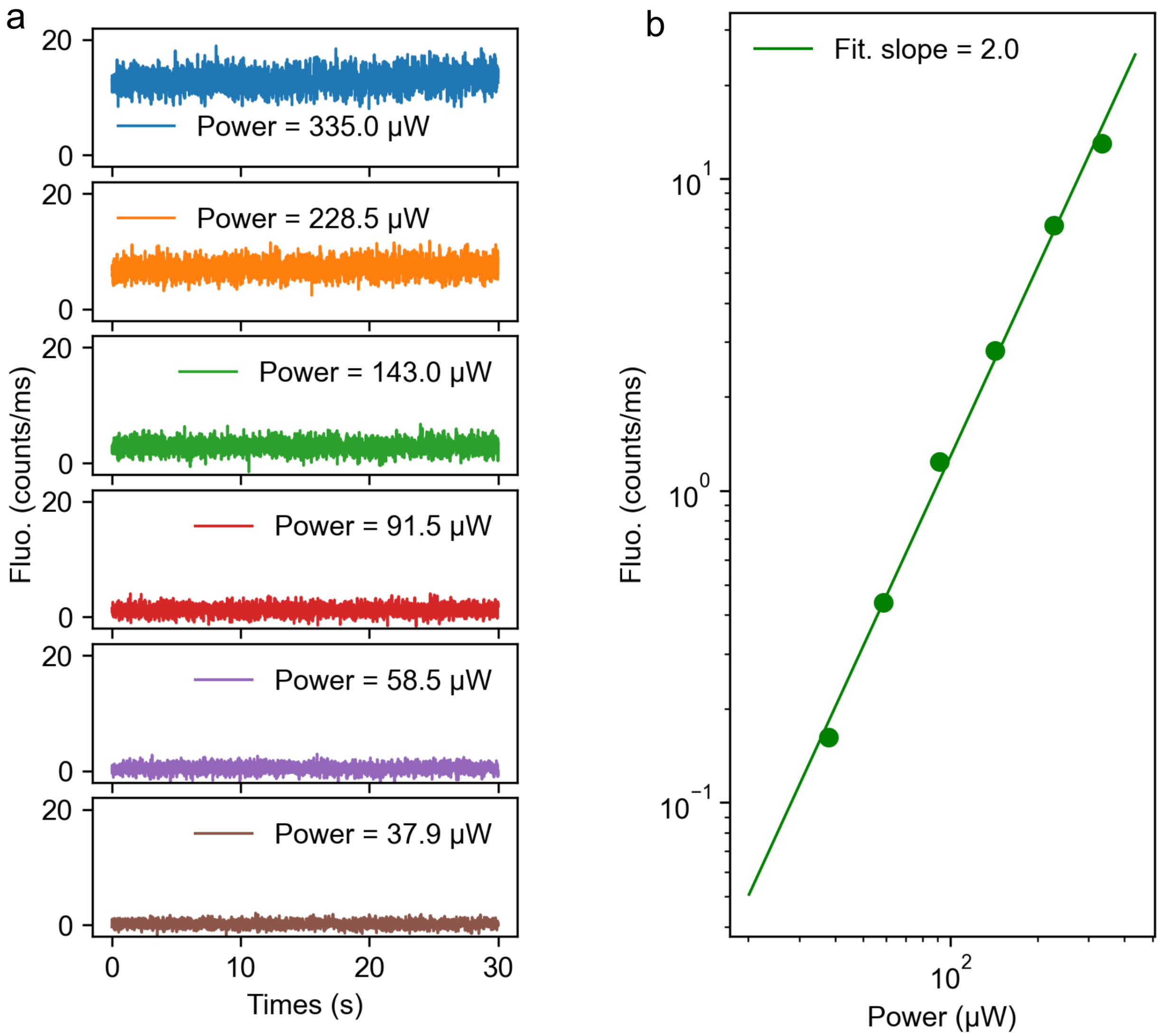

Figure S4: Power dependence of the emission measured in ATTO 610 solution (3 μM). (a) Fluorescence time traces excited with different laser powers. (b) Averaged fluorescence intensity as a function of the excitation power.

the scattered laser at high power, we first performed power-dependent measurements in a blank solution without any dyes in it. Time traces were recorded again after replacing the water with an ATTO 610 solution at the same conditions of the blank experiments. The fluorescence signals from the molecules, therefore, can be represented by intensity time traces with molecules after subtraction of the mean intensity recorded in the blank solution (see Figure S4a).

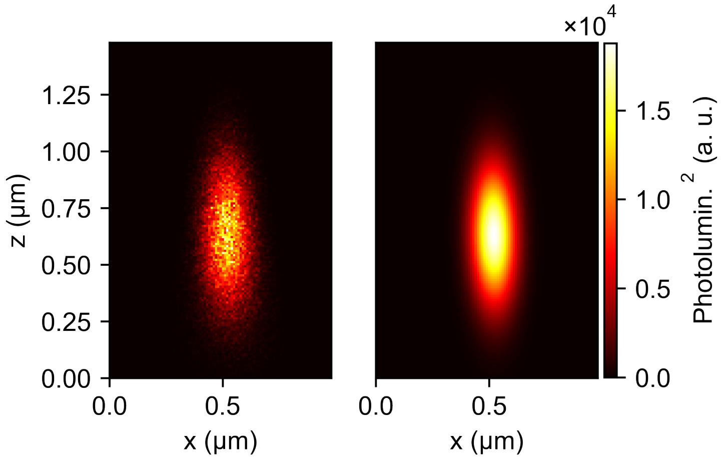

Figure S5: xz section of the two-photon excitation point spread function measured with a gold nanorod. Left column, the xz section of the square of one-photon excited photoluminescence exited by pulse laser. Right column, two-dimensional Gaussian fit of the PSF, from which we get the dimensions of the PSF, $\omega_x = 0.14$ μm and $\omega_z = 0.42$ μm and the PSF volume of 0.015 fL.

Figure S4b shows the quadratic dependence of the average fluorescence intensity measured in solution on the excitation laser power, indicating that the fluorescence arose from two-photon excitation. The overall recorded signals can be simply considered as coming from the contributions of the molecules in the confocal volume.

We calculated the size of the confocal volume for two-photon excitation by measuring the confocal point spread function (PSF) of the setup. We first scanned sectional one-photon excited luminescence images of a single GNR immersed in water excited with the pulse laser. The PSF of the setup for two-photon excitation can expressed as the square of the PSF for one photon. Shown in Figure S5, we estimated the two-photon excitation confocal volume to

be 0.015 fL, hence we estimate that about 20 molecules are in the upper half of the confocal volume for dye concentration of 3 μM.

Figure S6 shows the concentration dependence study of the fluorescence burst frequency at three different concentrations (3 nM, 10 nM and 30 nM) of ATTO 610 on the same nanostructure. Afterwards, the sample was cleaned thoroughly with de-ionized water to remove ATTO 610 dye molecules. Then, a control measurement was done at 0 nM using de-ionized water only. We chose the threshold value of 1.3 count/ms based on the maximum background value at 0 nM concentration (Figure S6a, top). We see 14, 52 and 159 bursts for the respective concentration of 3 nM, 10 nM and 30 nM and for the acquisition time of 300 seconds. The log-log plot in Figure S6b shows a linear relationship between the burst frequency and the concentration (red dots and red line in Figure S6b). Varying the threshold value slightly above or below doesn't change the relationship drastically, as shown in the Figure S6b. The blue dots and blue line correspond to the threshold value of 2.0 counts/ms and green dots and green line correspond to the threshold value of 1.0 counts/ms. This linear relationship between the burst frequency and the dye concentration provides an independent proof that the observed fluorescence signals are truly single-molecule events and are not caused by molecular aggregates.

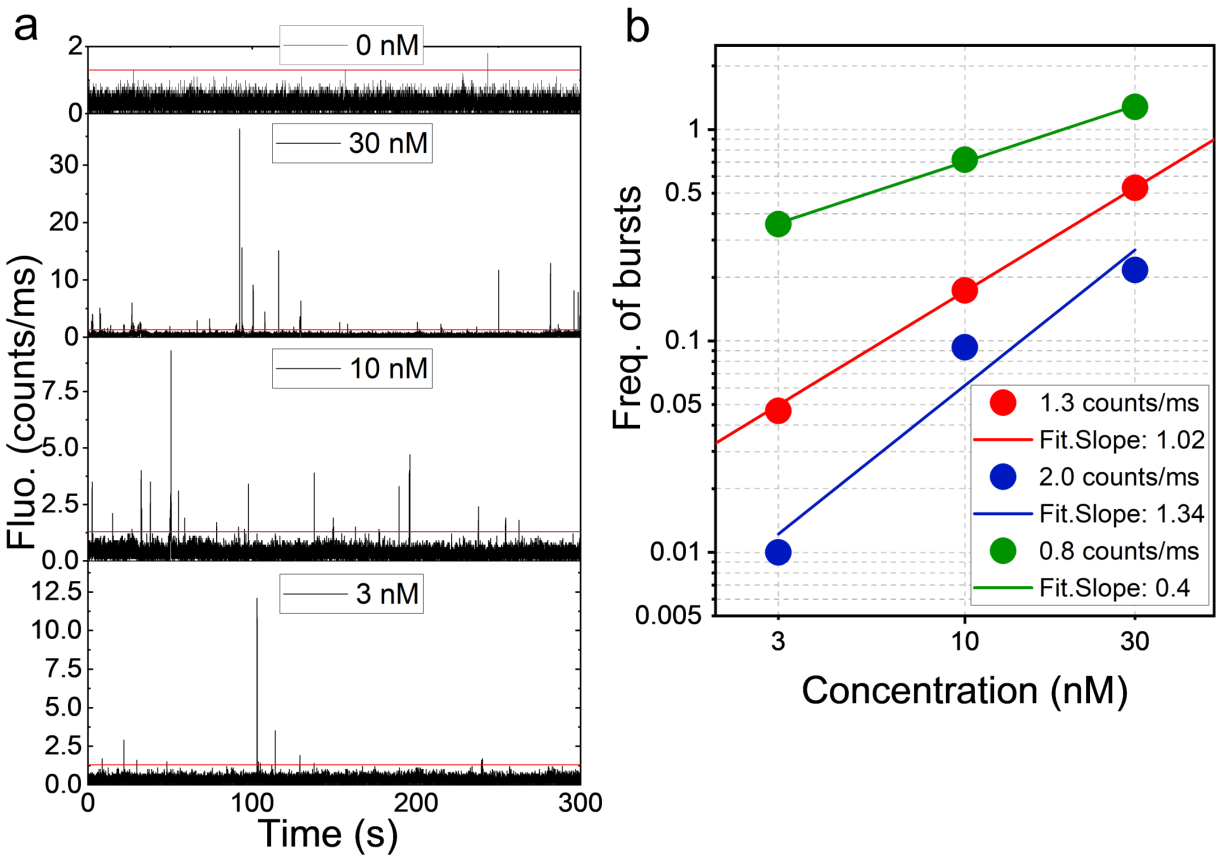

Figure S6: Concentration dependence of fluorescence burst frequency (a) Time traces recorded on the same nanostructure at three different ATTO 610 concentrations of 3 nM, 10 nM and 30 nM. The red line shows the upper threshold of 1.3 counts/ms obtained from the time trace at 0 nM (b) Log-log plot showing linear dependence of the frequency of bursts on the concentration of ATTO 610 for different threshold values of 1.3 counts/ms (red dots and red line), 2.0 counts/ms (blue dots and blue line) and 1.0 counts/ms (green dots and green line).

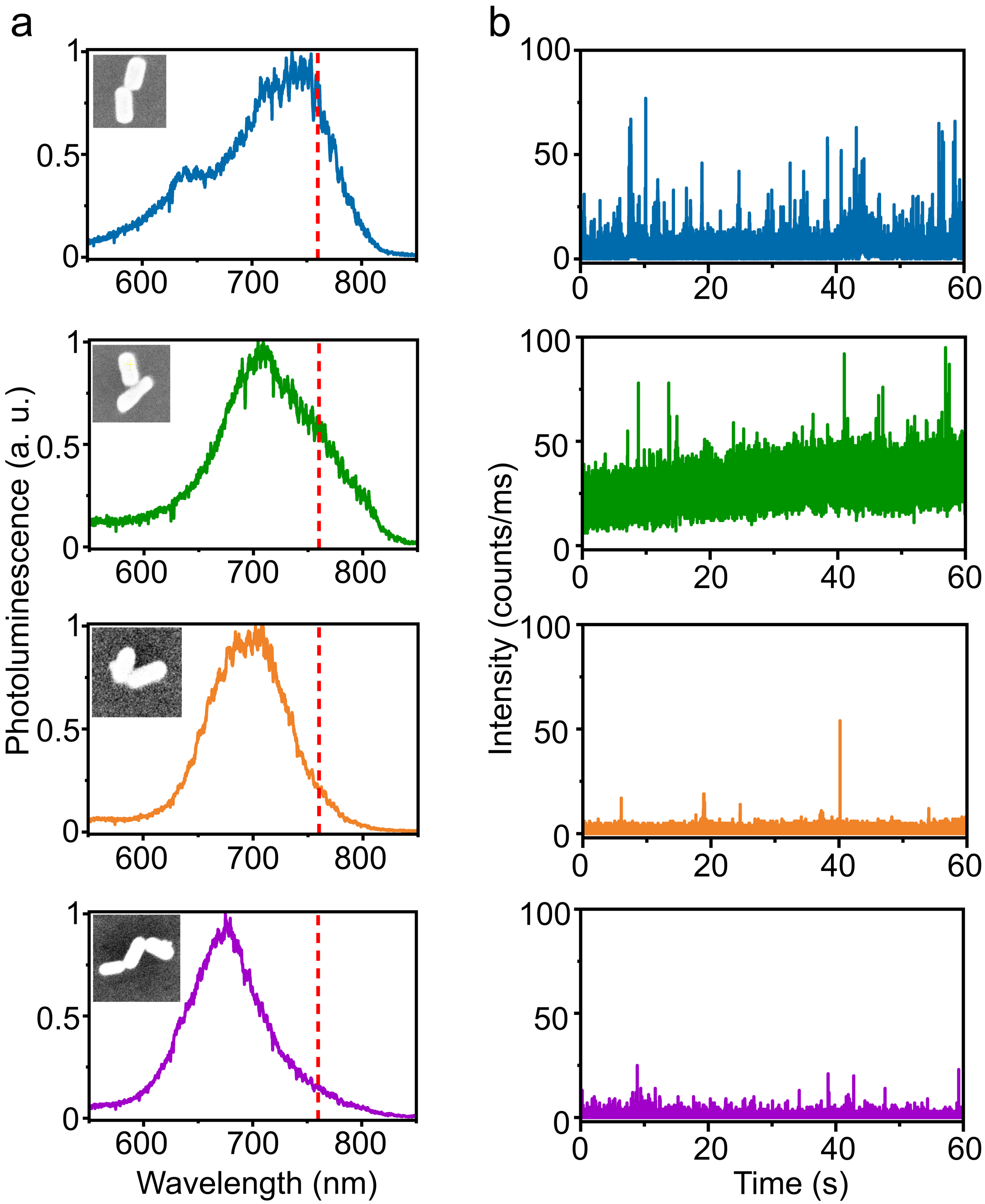

Figure S7: Two-photon-excited single-molecule fluorescence enhancement. (a) One-photon-excited luminescence spectra taken on four different structures made of gold nanorods, acquired under excitation by a circularly polarized 532 nm CW laser. Inset shows the SEM images of the structures. (b) The respective intensity traces taken in the presence of 20 nM ATTO 610 dyes, excited by a femtosecond laser at the wavelength of 760 nm (red dashed line in a)and at the power of ~ 2 μW. The binning time was set as 10 ms.

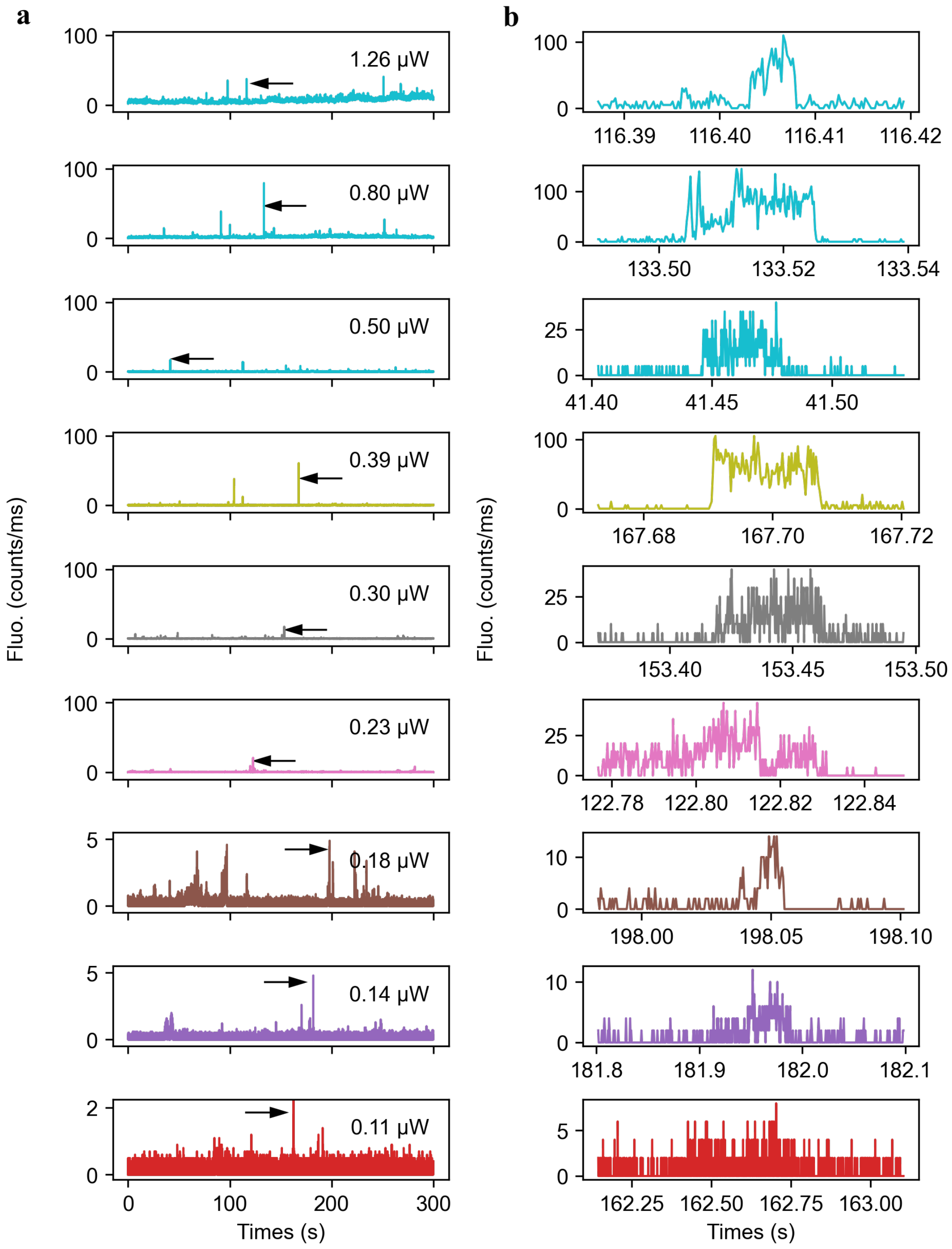

Figure S8: Single-molecule fluorescence enhancement under two-photon-excitation (a) Emission time trace (10 ms/bin) as a function of excitation power recorded on a gold nanorod self-assembled nanostructure. The particle was immersed in a solution of ATTO 610 with the concentration of 30 nM. (b) The zoomed views of the highest bursts of the time trace indicated by the black arrows in (a). The binning time was set as 0.5 ms for the zoomed-in time traces excited below the power of 0.2 μW, while for the traces excited above 0.2 μW, the binning time was set as 0.2 ms. The single-step intensity changes of these bursts confirm that the enhanced fluorescence signals are stemming from single molecules.

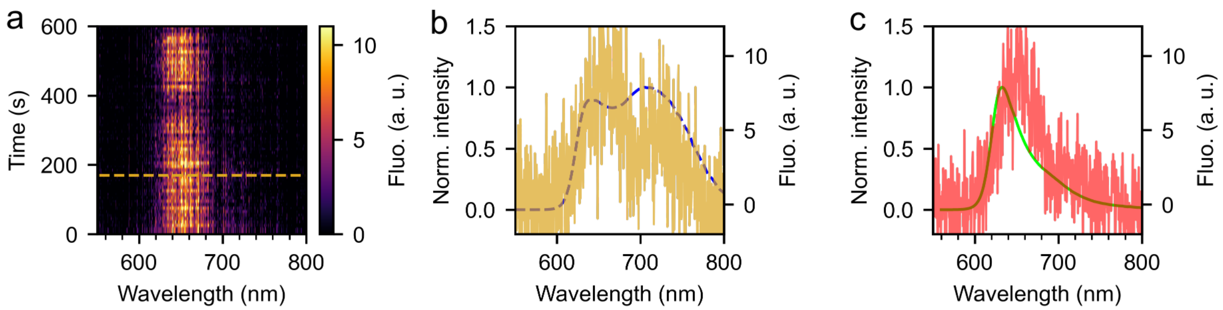

Figure S9: Emission spectra of ATTO 610 under two-photon-excitation enhanced by gold nanorod assembly. (a) Real-time spectra on gold nanorod dimer with the presence of 100 nM ATTO 610 solution. (b) Comparison of measured single-molecule spectrum (orange, corresponding to the recorded time of the orange dashed line in a) with the simulated enhanced spectra (blue dashed). For both spectra, we see the selected enhancement of the emission in the vibrational band of around 730 nm by the plasmon structure. (c) The measured spectra averaged over the recording time (red) and the spectrum of free ATTO 610 dye in solution (green line). The disappearance of the enhanced peak of the integrated spectra at around 730 nm, compared to (b), may stem from the averaging effect over different molecules having different positions or orientations with respect to the gap of the gold nanorod dimer, since the stimulated emission enhancement of the emitters by the plasmonic structure is dependent on their relative positions and orientations.

Numerical simulations of two-photon-excited fluorescence enhancement

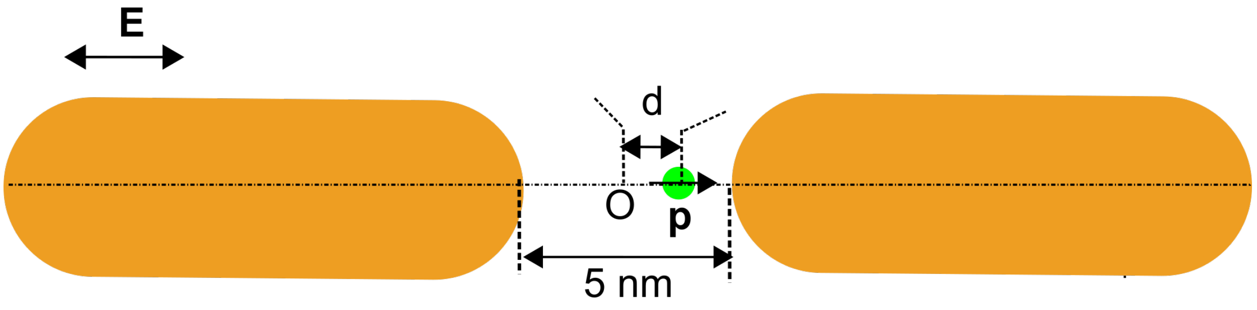

Figure S10: Scheme for the numerical simulations.

We performed numerical simulations for the two-photon-excited fluorescence enhancement under weak excitation, where the excitation enhancement and the emission enhancement can be treated separately.[S2] The scheme for the simulations is illustrated in Figure S10. For the sake of simplicity, we considered the dimer consisting of two identical GNRs with the longitudinal axes oriented in parallel, and separated by a gap of 5 nm. The size of

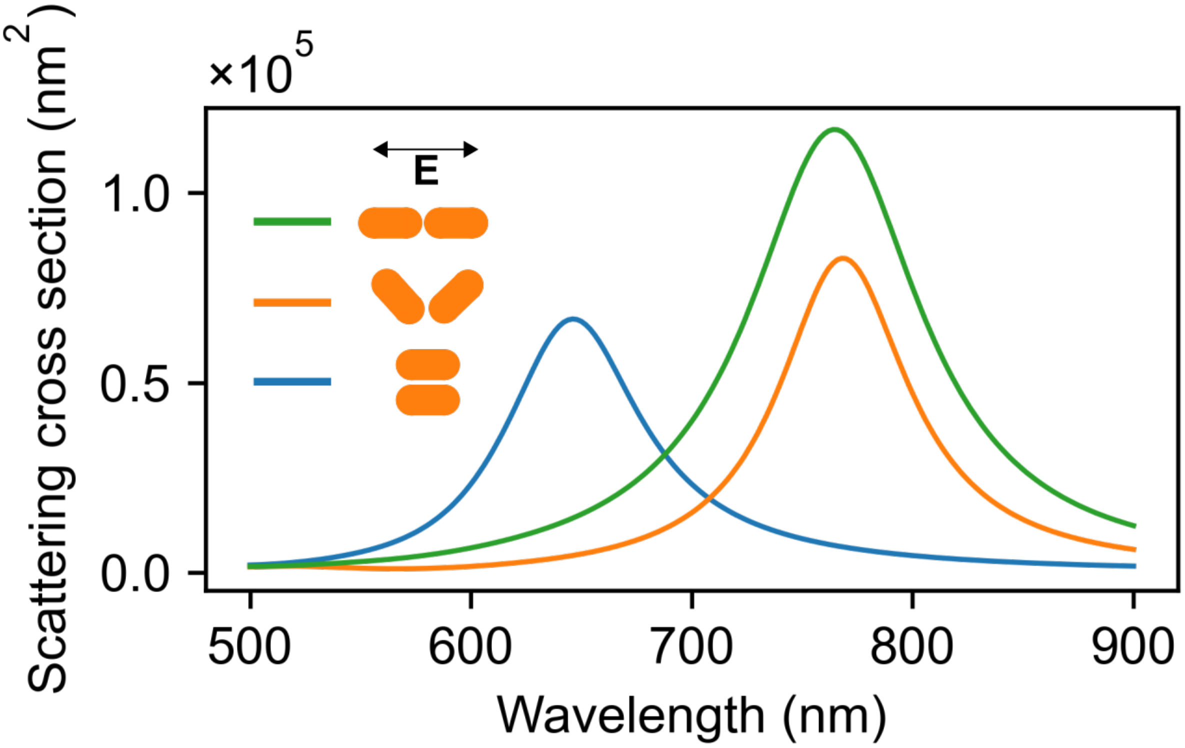

Figure S11: Scattering spectra of gold nanorod dimers with three different geometries having angles 180° (green), 90° (orange) and 0° (blue). The gap between the nanorods is 5 nm.

each GNR was set as 40 nm × 90 nm to guarantee a resonance at 760 nm. The ATTO 610 molecule was modeled as a radiative dipole with the position and dipole moment aligned along the longitudinal axis of the GNR dimer inside the gap. In the simulations, each GNR was modeled as a spherically capped cylinder. The dielectric permittivity for gold was taken from Johnson and Christy[S3] as used by many authors earlier,[S4,S5] and the refractive index of the ambient medium was taken as 1.33. Figure S10 shows the scattering spectra in the cases of three different angles between the two GNRs.

For two-photon-excited fluorescence, the excitation enhancement is related to the near-field enhancement :

$$\xi_{\mathrm{exc}}^{(2)} = |\mathbf{E}|^4/|\mathbf{E}_0|^4, \tag{S1}$$

where ($\mathbf{E}$) and $\mathbf{E}_0$ is the (near-)field (with) and without the gold nanorod dimer.

To evaluate the enhancement factor by the GNR dimer numerically, we applied a classical electrodynamics approach based on a boundary element method (SCUFF-EM)[S6,S7] to simulate the excitation and emission enhancement, respectively. To calculate the excitation enhancement, we excited the dimer structure with a plane wave with the polarization along the longitudinal axis of the dimer. The enhancement factor of the radiative rate (ξ_{rad}) and the additional non-radiative rate (K_{nr}) were calculated following the literature.[S8,S9] The expected emission spectrum enhanced by the dimer can expressed as[S10]

$$F(\omega) = \xi_{\mathrm{exc}}^{(2)} \cdot f(\omega) \cdot \xi_{\mathrm{rad}}(\omega) \cdot \frac{1/\eta_0}{<\xi_{\mathrm{rad}} + K_{\mathrm{nr}}/k_{\mathrm{r}}^0 - 1> + 1/\eta_0}, \tag{S2}$$

where k_{r}^0 is the intrinsic radiative rate of the dye, $f(\omega)$ is the normalized emission spectrum of ATTO 610 in solution, $< \cdots >$ represents the averaging over the emission spectrum of $f(\omega)$, η_0 is the intrinsic quantum yield of the molecule, which is 0.7 for ATTO 610. The overall enhancement can be written as

$$\xi_{\mathrm{total}} = \xi_{\mathrm{exc}}^{(2)} \cdot <\xi_{\mathrm{rad}}> \cdot \frac{1/\eta_0}{<\xi_{\mathrm{rad}} + K_{\mathrm{nr}}/k_{\mathrm{r}}^0 - 1> + 1/\eta_0}. \tag{S3}$$

References

(S1) Lu, X.; Punj, D.; Orrit, M. Controlled synthesis of gold nanorod dimers with end-to-end configurations. *RSC Advances* **2022**, *12*, 13464–13471.

(S2) Khatua, S.; Paulo, P. M. R.; Yuan, H.; Gupta, A.; Zijlstra, P.; Orrit, M. Resonant Plasmonic Enhancement of Single-Molecule Fluorescence by Individual Gold Nanorods. *ACS Nano* **2014**, *8*, 4440–4449.

(S3) Johnson, P. B.; Christy, R. W. Optical Constants of the Noble Metals. *Physical Review B* **1972**, *6*, 4370–4379.

(S4) Kong, X.-T.; Khosravi Khorashad, L.; Wang, Z.; Govorov, A. O. Photothermal Circular Dichroism Induced by Plasmon Resonances in Chiral Metamaterial Absorbers and Bolometers. *Nano Letters* **2018**, *18*, 2001–2008.

(S5) Fofang, N. T.; Park, T.-H.; Neumann, O.; Mirin, N. A.; Nordlander, P.; Halas, N. J. Plexcitonic Nanoparticles: Plasmonâˆ'Exciton Coupling in Nanoshellâˆ'J-Aggregate Complexes. *Nano Letters* **2008**, *8*, 3481–3487.

(S6) Reid, M. T. H.; Johnson, S. G. Efficient Computation of Power, Force, and Torque in BEM Scattering Calculations. *IEEE Transactions on Antennas and Propagation* **2015**, *63*, 3588–3598.

(S7) Homer Reid, M. T.; Johnson, S. G. Efficient Computation of Power, Force, and Torque in BEM Scattering Calculations. *ArXiv e-prints* **2013**, `http://github.com/homerreid/scuff-EM`.

(S8) Lu, X.; Ye, G.; Punj, D.; Chiechi, R. C.; Orrit, M. Quantum Yield Limits for the Detection of Single-Molecule Fluorescence Enhancement by a Gold Nanorod. *ACS Photonics* **2020**, *7*, 2498–2505.

(S9) Zhang, W.; Caldarola, M.; Lu, X.; Orrit, M. Plasmonic Enhancement of Two-Photon-Excited Luminescence of Single Quantum Dots by Individual Gold Nanorods. *ACS Photonics* **2018**, *5*, 2960–2968.

(S10) Ringler, M.; Schwemer, A.; Wunderlich, M.; Nichtl, A.; Kürzinger, K.; Klar, T. A.; Feldmann, J. Shaping Emission Spectra of Fluorescent Molecules with Single Plasmonic Nanoresonators. *Physical Review Letters* **2008**, *100*, 203002.
